# Effects of the Synbiotic Formulation EDC-HHA01 on Glucose Regulation in Adults with Type 2 Diabetes and Prediabetes: A Randomized, Placebo-Controlled Study

**DOI:** 10.3390/microorganisms14040749

**Published:** 2026-03-26

**Authors:** Gissel García, María del Carmen Campos, Josanne Soto, Antonio Diaz, Emilio Buchaca, Duniesky Martínez, Mirka Bernal, Viviana Escobar, Lais Rodríguez, Eduardo Valdés, Maricela Nuez, Noraika Domínguez, Liuvob Sichel, Raúl de Jesús Cano

**Affiliations:** 1 Pathology Department, Hermanos Ameijeiras Clinical and Surgical Hospital, Calle San Lázaro No. 701, Esquina a Belascoaín, La Habana 10400, Cuba; gisselgarcia2805@gmail.com; 2 Clinical Laboratory Department, Hermanos Ameijeiras Clinical and Surgical Hospital, Calle San Lázaro No. 701, Esquina a Belascoaín, La Habana 10400, Cuba; camposcast.carmen@hotmail.com (M.d.C.C.); josannesoto99@gmail.com (J.S.); mirka.bernal74@gmail.com (M.B.); 3 Statistical and Research Department, Hermanos Ameijeiras Clinical and Surgical Hospital, Calle San Lázaro No. 701, Esquina a Belascoaín, La Habana 10400, Cuba; adm12101972@gmail.com; 4 Internal Medicine Department, Hermanos Ameijeiras Clinical and Surgical Hospital, Calle San Lázaro No. 701, Esquina a Belascoaín, La Habana 10400, Cuba; emiliobuchacafaxas@gmail.com (E.B.); laysrodriguezamador@gmail.com (L.R.); 5 Research and Development Department, Center for Genetic Engineering and Biotechnology of Sancti Spíritus, Circunvalante Norte, Reparto Olivos 3, Sancti Spiritus 60200, Cuba; duniesky.martinez@cigb.edu.cu; 6 Nutrition Department, Hermanos Ameijeiras Clinical and Surgical Hospital, Calle San Lázaro No. 701, Esquina a Belascoaín, La Habana 10400, Cuba; vivianaescobardibut@gmail.com (V.E.); evaldesbencosme@gmail.com (E.V.); mariselanuezvilar@gmail.com (M.N.); 7 Endocrinology Department, Hermanos Ameijeiras Clinical and Surgical Hospital, Calle San Lázaro No. 701, Esquina a Belascoaín, La Habana 10400, Cuba; noraikadominguez@gmail.com; 8 Stellar Biotics, LLC, Rockleigh, 22 Paris Ave, Suite 209, Rockleigh, NJ 07647, USA; luba@stellarbiotics.com; 9 Biological Sciences Department, California Polytechnic State University, San Luis Obispo, CA 93405, USA; 10 Chauvel, LLC, San Luis Obispo, CA 93405, USA

**Keywords:** microbiome, prediabetes, type 2 diabetes mellitus, glycated hemoglobin (HbA1c), insulin resistance, metabolic endotoxemia, probiotics, randomized controlled trial

## Abstract

Microbiome-targeted interventions have shown promise for metabolic health, yet clinical evidence remains inconsistent, particularly across stages of metabolic disease. This study evaluated the metabolic effects, safety, and tolerability of EDC-HHA01, a microbiome-informed, non-pharmacologic intervention, in adults with prediabetes (PD) or Type 2 Diabetes (T2DM). In a randomized, double-blind, placebo-controlled clinical trial, participants received EDC-HHA01 or placebo for six months. The study was adequately powered (≥80%) for the primary endpoint. Outcomes included changes in glycated hemoglobin (HbA1c), indices of insulin resistance, markers of metabolic endotoxemia, safety-related laboratory parameters, and exploratory patient-reported measures. Analyses were stratified by metabolic status and background metformin use. In participants with PD, EDC-HHA01 supplementation was associated with a statistically and clinically meaningful reduction in HbA1c compared with placebo, supported by concordant improvements in fasting insulin, insulin resistance indices, and reductions in endotoxemia markers. In participants with T2DM, changes were directionally similar but attenuated and did not reach statistical significance. The intervention was well tolerated, with no serious adverse events, high adherence, and no clinically relevant adverse changes in renal or lipid parameters. Exploratory patient-reported outcomes indicated favorable acceptability but were not interpreted as efficacy endpoints. EDC-HHA01 was associated with biologically coherent, stage-dependent metabolic effects, most evident in PD. These findings support further investigation of microbiome-informed strategies as metabolic support in early-stage dysregulation.

## 1. Introduction

Diabetes is a complex, chronic metabolic disorder that requires continuous medical care and multifactorial risk-reduction strategies extending beyond glycemic control alone [1]. By 2022, the global prevalence of diabetes among adults aged 18 years and older had doubled since 1990, increasing from approximately 7% to 14%. Despite this rise, more than half of adults aged 30 years or older living with PD were not receiving pharmacological treatment, with treatment coverage particularly limited in low- and middle-income countries, highlighting persistent disparities in access to care [2].

Type 2 diabetes (T2DM), the most prevalent form of diabetes, is typically diagnosed based on elevated fasting plasma glucose (≥7.0 mmol/L or 126 mg/dL) and/or impaired glucose tolerance (≥11.1 mmol/L following oral glucose challenge) [3]. Prior to meeting diagnostic thresholds, many individuals experience prediabetes (PD), a transitional metabolic state characterized by impaired glucose regulation and increased risk of progression to T2DM and associated cardiometabolic complications [4]. While standard management of T2DM includes lifestyle modification and oral antihyperglycemic agents such as metformin, achieving durable glycemic control remains challenging for many patients, even with appropriate therapy.

Increasing evidence implicates host–microbial interactions as important contributors to metabolic regulation and the progression of metabolic disease [5,6]. Microbial communities and their fermentation-derived metabolites influence host glucose metabolism, lipid handling, immune signaling, intestinal barrier integrity, and systemic inflammatory tone [7]. Dysregulation of these processes has been associated with insulin resistance, metabolic endotoxemia, and chronic low-grade inflammation—features common to both T2DM and prediabetic states [6,8,9]. Importantly, these pathways may retain greater physiological plasticity during earlier stages of metabolic impairment, suggesting that interventions targeting metabolic regulation may be more effective before advanced disease is established [10,11,12].

In 2022, Cuba conducted its first human clinical study evaluating the glucose-regulating effects of the synbiotic formulation BiotiQuest Sugar Shift^®^ in patients with T2DM [13]. That study demonstrated improvements in fasting and postprandial glucose, lipid parameters, and markers of endotoxemia, although HbA1c levels remained unchanged following three months of intervention. A subsequent six-month pilot study reported broader metabolic benefits, including a reduction in HbA1c. Notably, exploratory analyses suggested that individuals with less advanced metabolic dysregulation, including those with PD, exhibited more consistent and pronounced metabolic responses. These findings informed the rational development of a next-generation synbiotic formulation designed to more directly support metabolic regulation, immune–metabolic balance, and inflammatory control—processes relevant to both T2DM and PD [13,14].

The formulation evaluated in the present study, designated EDC-HHA01, is a scientifically designed synbiotic developed through genetic strain profiling and community-level metabolic modeling.

PD represents a critical opportunity for metabolic risk mitigation, as regulatory pathways governing glucose homeostasis, inflammatory tone, and insulin sensitivity may remain partially intact and responsive to non-pharmacological intervention [8,11,15]. Accordingly, the present study conceptualizes synbiotic supplementation not solely as an adjunctive strategy in established T2DM, but also as a potential risk-modifying intervention in individuals with PD, aimed at supporting metabolic regulation and delaying disease progression.

### Study Hypothesis

The primary hypothesis of this study is that daily supplementation with the synbiotic formulation EDC-HHA01 will result in clinically meaningful improvements in glucose regulation compared with placebo. It is further hypothesized that the magnitude and consistency of response will be greater in individuals with PD than in those with established T2DM, reflecting greater metabolic plasticity at earlier stages of dysregulation. Secondary hypotheses posit that improvements in glycemic measures will be accompanied by favorable changes in metabolic and inflammatory biomarkers, supporting a biologically plausible mechanism of action.

## 2. Materials and Methods

### 2.1. Ethical Considerations

The study was designed as a randomized, double-blind, placebo-controlled human clinical trial to evaluate the safety and probiotic potential of the synbiotic formulation EDC-HHA01 in adults diagnosed with T2DM or PD. The study protocol was reviewed and approved by the Ethics Committee of the Hermanos Ameijeiras Clinical and Surgical Hospital (approval number NH24ILH0040; 1 April 2024), the National Institute of Nutrition of Cuba (PI45872/24), and the Cuban Ministry of Public Health.

All study procedures were conducted in accordance with Good Clinical Practice (GCP) guidelines and the ethical principles set forth in the Declaration of Helsinki [16]. Written informed consent was obtained from all participants prior to enrollment.

The trial was registered in the Cuban Public Registry of Clinical Trials (Registro Público Cubano de Ensayos Clínicos, RPCEC) under registration number RPCEC00000441 on 30 May 2024.

### 2.2. Sample Size

Sample size estimation was informed by prior clinical experience at the Hermanos Ameijeiras Clinical and Surgical Hospital involving patients with T2DM receiving probiotic interventions [14]. To ensure adequate statistical reliability while maintaining feasibility, a target sample size of approximately 100 participants was selected.

An initial estimate indicated that a minimum of 94 participants would be required to detect clinically meaningful differences between groups with a 95% confidence level. To account for potential attrition and ensure balanced representation of diagnostic subgroups, the final target enrollment was set at 100 participants. The selected sample size was informed by prior clinical experience and was deemed sufficient to provide approximately 80% power to detect moderate, clinically meaningful differences in primary metabolic outcomes between treatment groups, assuming typical variability observed in similar populations [17].

Participants were randomly assigned in a 1:1 ratio to receive either the EDC-HHA01 formulation or placebo, resulting in two study arms of 50 participants each. Within each arm, enrollment was stratified by baseline diagnosis to include approximately equal numbers of individuals with T2DM and PD (25 per diagnostic subgroup).

This sample size was considered sufficient to support the planned primary and secondary analyses while accommodating expected variability in metabolic outcomes and participant retention.

### 2.3. Study Design and Participants

This study was conducted as a randomized, double-blind, placebo-controlled clinical trial at the Hermanos Ameijeiras Clinical and Surgical Hospital (HHA) in Havana, Cuba. Participant recruitment began with the enrollment of the first participant on 10 June 2024, and concluded with the enrollment of the final participant on 2 December 2024.

The intervention period lasted six months, with participants receiving study product from January through June 2025. Participants were followed through scheduled clinical consultations throughout the intervention period and for an additional three months after completion of supplementation, with follow-up concluding in September 2025.

Eligible participants were adults aged 18 years or older, of either sex, with a confirmed clinical diagnosis of T2DM or PD, managed with oral antihyperglycemic therapy and/or dietary intervention alone. Individuals receiving insulin therapy were not eligible for inclusion. All participants provided written informed consent prior to enrollment.

Participants were required to be clinically stable, free of acute illness, and not consuming probiotic or synbiotic supplements at the time of enrollment. Exclusion criteria included type 1 PD or other specific forms of PD, insulin therapy, chronic kidney disease, active or uncontrolled comorbid conditions (including untreated endocrine disorders, acute infections, major depressive disorders, or malignant disease), pregnancy or lactation, substance abuse, or cognitive or psychological conditions that could impair adherence or informed consent, as assessed by the principal investigator. Individuals participating in other clinical trials or who had received symptom-altering interventions within the preceding three months were also excluded.

Randomization was performed using EpiData version 3.1 software [18], which generated a computer-based random allocation sequence assigning participants in a 1:1 ratio to receive either the EDC-HHA01 formulation or placebo. Randomization was stratified by baseline diagnosis (T2DM or PD) to ensure balanced representation across treatment arms. Allocation concealment was maintained by dispensing the investigational product and placebo in identical capsules and packaging. Investigators, study staff, and participants remained blinded to treatment assignment throughout the study.

Inclusion criteria required participants to be clinically stable, free of acute illness, and not consuming probiotic or synbiotic supplements at the time of enrollment. Exclusion criteria included type 1 diabetes or other specific forms of PD insulin therapy, chronic kidney disease, active or uncontrolled comorbid conditions (including untreated endocrine disorders, acute infections, depressive disorders, or malignant disease), pregnancy or lactation, substance abuse, or cognitive or psychological conditions that could impair adherence or informed consent, as assessed by the principal investigator. Individuals enrolled in other clinical trials or who had received symptom-altering interventions within the preceding three months were also excluded.

A total of 92 participants were enrolled and randomized, comprising 40 participants with PD and 52 with T2DM. All randomized participants were included in the intention-to-treat population. Following attrition and loss to follow-up, data from 38 participants in the EDC-HHA01 group (20 with T2DM and 18 with PD) and 37 participants in the placebo group (21 with T2DM and 16 with PD) were available for analysis of the primary outcome. Study adherence, assessed through capsule count, exceeded 94% across all groups, and no protocol deviations affecting the primary outcome analyses were identified.

**Figure 1:**
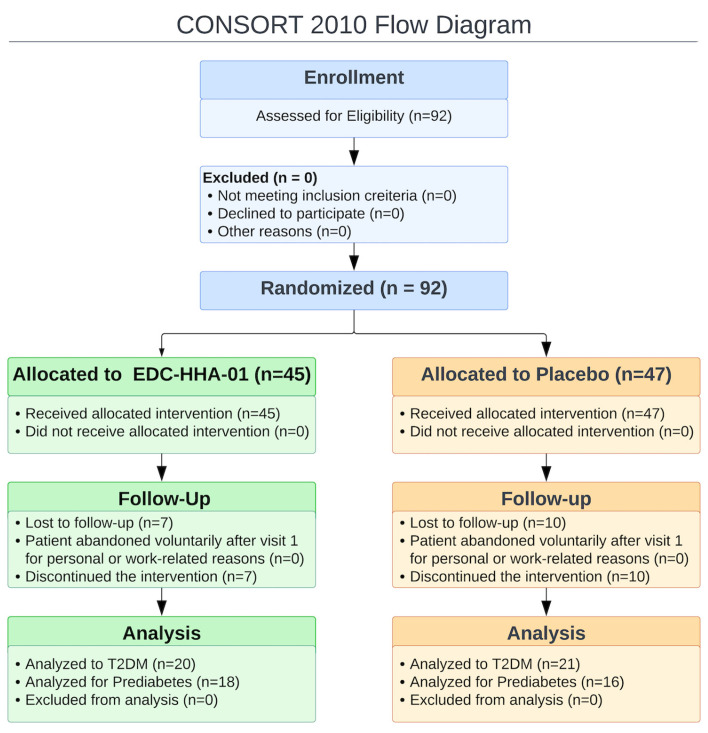
CONSORT [19] flow diagram of recruitment and retention throughout the study.

Baseline demographic characteristics showed no significant between-group differences (Table 1, all *p* > 0.05). Baseline metabolic parameters are presented in Supplementary Table S1 and showed balanced distribution across intervention and placebo groups within diagnostic strata. Participant flow throughout the study is illustrated in the CONSORT flow diagram (Figure 1).

#### Concomitant Care

Participants continued to receive usual medical care throughout the study period. Background medications, including stable glucose-lowering therapies such as metformin where applicable, were permitted provided dosing remained unchanged. No protocol-directed changes to concomitant medications, diet, or physical activity were mandated during the study, and no new glucose-lowering therapies were initiated as part of the intervention. Concomitant care was comparable between the EDC-HHA01 and placebo groups.

### 2.4. Development and Composition of the Synbiotic Formulation (EDC-HHA01)

The investigational product evaluated in this study, designated EDC-HHA01, is a synbiotic formulation developed through a rational, systems-based design process integrating probiotic organisms, prebiotic substrates, and a defined metabiotic component. The formulation is commercially available as Advanced Daily; however, the study denomination is used throughout this manuscript to preserve scientific neutrality.

Formulation development was guided by the objective of supporting metabolic and glycemic regulation through coordinated microbial functions rather than reliance on the activity of individual strains. Design considerations integrated genomic annotation, metabolic pathway analysis, and in silico modeling as decision-support tools to inform strain selection and formulation logic, while empirical evidence from peer-reviewed literature and biological plausibility guided final design decisions.

Candidate probiotic organisms were selected based on established safety history, suitability for encapsulated delivery, and reported involvement in host-relevant metabolic, inflammatory, and gut barrier-associated pathways. Genome-scale metabolic reconstructions were generated using the ModelSEED v2 [20] framework to assess pathway completeness, substrate utilization breadth, and potential functional complementarity among candidate organisms. Community-level modeling was used internally to guide formulation decisions; however, modeling architectures beyond strain-level reconstruction are proprietary.

The final probiotic consortium comprises six bacterial species representing the genera *Bacillus*, *Bifidobacterium*, and *Lactobacillus*: *Bacillus coagulans*, *Bifidobacterium bifidum*, *Limosilactobacillus reuteri*, *Lactiplantibacillus plantarum*, *Lacticaseibacillus rhamnosus*, and *Lacticaseibacillus casei*. Strain-level identifiers were retained internally throughout development to ensure traceability across manufacturing, quality control, and clinical evaluation but are not disclosed here to protect proprietary genomic assets. The consortium was designed to balance robustness and functional diversity by combining spore-forming and non–spore-forming taxa while minimizing unnecessary functional redundancy.

In addition to live microorganisms, the formulation incorporates both prebiotic [21] and metabiotic [22] components to enhance functional stability and reduce reliance on probiotic engraftment alone. The prebiotic fraction includes inulin derived from chicory root, selected to support fermentative activity of bifidobacteria and lactobacilli and to promote microbial metabolic outputs associated with short-chain fatty acid–relevant pathways. Microcrystalline cellulose is included as a formulation excipient and insoluble fiber but was not intended to serve as a targeted prebiotic substrate.

The metabiotic component consists of an enzymatic lysate of *Lactobacillus delbrueckii* [23], produced via lysozyme-mediated cell wall hydrolysis. This process yields non-viable microbial cellular constituents while preserving structurally and functionally relevant bioactive components. Inclusion of the metabiotic component was intended to provide microbial-derived signaling molecules independent of in vivo replication, thereby increasing robustness of functional delivery across individuals with heterogeneous baseline microbiological states. Detailed manufacturing specifications for the lysate are proprietary.

The formulation is labeled to provide approximately 15 × 10^9^ colony-forming units (CFU) per capsule. In the present clinical study, participants received two capsules daily, corresponding to an administered dose of approximately 30 × 10^9^ CFU per day. Each capsule also contained the prebiotic and metabiotic components described above, along with standard excipients required for capsule stability and delivery.

Mechanistic considerations described in this section represent the biological rationale underlying formulation development and should be interpreted as hypothesis-generating rather than as direct mechanistic evidence derived from the clinical outcomes reported in this study.

### 2.5. Intervention and Compliance

Participants were randomized to receive either the EDC-HHA01 formulation or a matching placebo in a double-blind manner. The intervention period lasted six months. Participants in both study arms were instructed to consume one capsule every 12 h (twice daily) throughout the study duration.

The investigational product, EDC-HHA01, was manufactured in accordance with Good Manufacturing Practices (GMP) by Hermanos Ameijeiras Clinical and Surgical Hospital. Each capsule contained a total microbial consortium concentration of 1.48 × 10^10^ colony-forming units (CFU), composed of seven GRAS-classified bacterial strains: *Bacillus coagulans* (20 mg), *Bifidobacterium bifidum* (30 mg), *Limosilactobacillus reuteri* (30 mg), *Lactiplantibacillus plantarum* (45 mg), *Lacticaseibacillus rhamnosus* (45 mg), *Lacticaseibacillus casei* (45 mg), and *Lactobacillus delbrueckii* enzymatic lysate (10 mg). Each capsule also contained 100 mg of inulin as a prebiotic carrier.

Placebo capsules were indistinguishable in appearance, weight, and packaging from the active formulation and contained microcrystalline cellulose as the inert excipient. Placebo capsules were manufactured and packaged using the same procedures and materials as the active product to maintain blinding.

Both EDC-HHA01 and placebo capsules were dispensed in sealed foil packaging and provided to participants at two scheduled visits: baseline (week 0) and mid-intervention (week 12), corresponding to routine clinical assessments and sample collection. Participants were instructed to return unused capsules at follow-up visits, and adherence was monitored through capsule counts and participant self-report.

### 2.6. Sample Collection and Study Procedures

Participants underwent clinical evaluation, supplement administration, and biological sample collection at predefined 12-week intervals throughout the six-month intervention period (baseline, week 12, and month 6). Blood samples collected at Week 12 served safety monitoring and compliance verification purposes. Comprehensive endpoint analyses were prespecified for baseline and month 6; intermediate data will be reported in a planned comparative study with prior symbiotic interventions.

Venous blood samples were obtained by standard venipuncture, labeled with a unique participant identification code, and processed within one hour of collection. Samples were centrifuged, aliquoted, and stored under controlled conditions to preserve integrity for downstream biochemical analyses.

All clinical and laboratory data were recorded in a secure, password-protected database accessible only to authorized study and clinical personnel. The Hermanos Ameijeiras Clinical and Surgical Hospital (HHA) oversaw data security and management of the information technology infrastructure supporting the study.

Health-related quality of life and safety were assessed using the Semi-Structured Interview for Assessing sample size of Probiotics and Nutritional Supplements Survey [24], administered at baseline and at study completion. The primary clinical outcome was glycated hemoglobin (HbA1c). Secondary outcomes included fasting and postprandial glucose, total cholesterol, HDL cholesterol, LDL cholesterol, triglycerides, insulin, homeostatic model assessment indices (HOMA-IR and HOMA-β), and serum lipopolysaccharide (LPS) levels.

### 2.7. Study Outcomes

#### 2.7.1. Primary Outcome

The primary efficacy outcome was the change in glycated hemoglobin (HbA1c) from baseline to 6 months. HbA1c was selected a priori as the primary endpoint because it reflects integrated glycemic exposure over approximately 8–12 weeks and is less susceptible to short-term behavioral or dietary fluctuations than fasting or postprandial glucose measurements [25].

#### 2.7.2. Key Mechanistic (Exploratory) Outcome

A key mechanistic exploratory outcome was the change in circulating serum lipopolysaccharide (LPS) concentrations over the six-month intervention period. LPS was evaluated as a marker of systemic endotoxemia and gut barrier-related metabolic inflammation, given its established role in insulin resistance, low-grade chronic inflammation, and dysglycemia [26]. Assessment of LPS was intended to explore whether modulation of gut-derived endotoxin burden could provide a biological link between probiotic intervention and downstream metabolic effects.

Circulating LPS concentrations were measured using two independent analytical platforms to enhance robustness and reduce assay-specific bias. A Limulus amebocyte lysate (LAL)-based assay was performed using a commercial kit supplied by GenScript Biotech Corporation (Piscataway, NJ, USA). In parallel, LPS concentrations were quantified using an enzyme-linked immunosorbent assay (ELISA) (Human Lipopolysaccharides ELISA Kit, EK711143; AFG Bioscience, Northbrook, IL, USA), following the manufacturers’ instructions.

For ELISA-based measurements, all samples were analyzed in duplicate under standardized laboratory conditions using aseptic techniques to minimize contamination. Optical density readings were obtained using a SpectraMax iD50, calibrated microplate reader (Molecular Devices, San Jose, CA, USA), and LPS concentrations were calculated from standard curves generated for each assay run. Quality control procedures were applied in accordance with manufacturer recommendations to ensure assay precision and reproducibility.

Given the known variability and distributional challenges associated with circulating LPS measurements, analyses of LPS outcomes were conducted on an exploratory basis. LPS data were interpreted as supportive mechanistic evidence linking probiotic intervention to metabolic outcomes, rather than as confirmatory efficacy endpoints.

#### 2.7.3. Secondary Outcomes

Exploratory analyses included evaluation of mechanistic biomarkers and patient-reported outcomes related to gastrointestinal tolerability, perceived metabolic benefits, and overall well-being. Prespecified subgroup analyses were conducted by baseline diagnostic category (PD vs. T2DM) and background metformin use to explore potential stage-dependent and treatment-context-dependent effects.

Within-subject changes in LPS were combined into a robust composite endotoxemia index using median and median absolute deviation (MAD) standardization across analytical platforms. This composite index was intended to provide a distributionally robust measure of endotoxin burden and was interpreted as a supportive biological correlate of metabolic and glycemic outcomes rather than as a confirmatory efficacy endpoint.

Patient-reported outcomes were collected using a structured questionnaire assessing gastrointestinal symptoms, appetite regulation, perceived energy, psychological well-being, and general health status. Responses were recorded on a 5-point Likert scale and analyzed as exploratory measures intended to characterize tolerability, acceptability, and patient experience rather than therapeutic efficacy.

#### 2.7.4. Exploratory and Subgroup Analyses

Exploratory analyses included markers of metabolic endotoxemia, and patient-reported outcomes related to gastrointestinal tolerability, perceived metabolic benefits, and overall well-being. Prespecified subgroup analyses were conducted by baseline diagnostic category (PD vs. T2DM) and background metformin use to explore potential stage-dependent and treatment-context-dependent effects.

Markers of metabolic endotoxemia were assessed using circulating lipopolysaccharide (LPS) concentrations measured by two independent analytical platforms (GeneScript/LAL-based assay and ELISA-based assay (AFG Bioscience, Northbrook, IL, USA). Within-subject changes in LPS were combined into a robust composite endotoxemia index using median and median absolute deviation (MAD) standardization across platforms. This composite index was intended to provide a distributionally robust measure of endotoxin burden and was interpreted as a supportive biological correlate of metabolic and glycemic outcomes rather than as a confirmatory efficacy endpoint.

Patient-reported outcomes were collected using a structured questionnaire assessing gastrointestinal symptoms, appetite regulation, perceived energy, psychological well-being, and general health status. Responses were recorded on a 5-point Likert scale and analyzed as exploratory measures intended to characterize tolerability, acceptability, and patient experience rather than therapeutic efficacy.

### 2.8. Clinical Chemistry Analyses

Blood samples were collected at three predefined time points: baseline (one week prior to intervention), mid-intervention (week 12), and at the end of the study period (month 6). Glycated hemoglobin (HbA1c) was measured in whole blood collected into K_3_-EDTA tubes and analyzed immediately using a validated automated analyzer (Cobas 6000 modular system; Roche Diagnostics, Indianapolis, IN, USA), in accordance with the manufacturer’s instructions.

For additional clinical chemistry assessments, venous blood was collected into serum-separating tubes without anticoagulant. Samples were allowed to clot at room temperature and subsequently centrifuged to obtain serum. Serum concentrations of fasting glucose, lipid parameters, and other biochemical markers were determined using standardized automated immunochemical and enzymatic assays (Cobas 600 platform; Roche Diagnostics).

All analyses were performed by trained laboratory personnel following established quality control procedures, including routine calibration and internal controls, to ensure analytical accuracy and reproducibility.

#### 2.8.1. Serum Lipopolysaccharide (LPS) Measurement

Serum concentrations of lipopolysaccharide (LPS) were quantified using a commercially available enzyme-linked immunosorbent assay (ELISA) kit (Human Lipopolysaccharides ELISA Kit, EK711143, AFG Bioscience LLC, Northbrook, IL, USA), following the manufacturer’s instructions. All samples were analyzed in duplicate to ensure analytical reliability.

Assays were performed under standardized laboratory conditions using aseptic techniques to minimize contamination. Optical density measurements were obtained using a calibrated microplate reader, and LPS concentrations were calculated from standard curves generated for each assay run. Quality control procedures were applied in accordance with the manufacturer’s recommendations to ensure assay precision and reproducibility.

#### 2.8.2. HOMA-IR and HOMA-β Index Calculations

Insulin resistance and pancreatic β-cell function were assessed using the Homeostasis Model Assessment indices HOMA-IR and HOMA-β. Fasting blood samples were obtained following an overnight fast of at least 8 h to ensure reliable baseline measurements. To standardize post-fasting conditions across participants, a controlled breakfast consisting of an energy bar appropriate for diabetic status was provided prior to subsequent assessments.

Serum insulin concentrations were quantified using a validated immunoassay, while fasting glucose levels were determined by enzymatic methods using an automated clinical chemistry analyzer (Cobas 6000 modular system; Roche Diagnostics, Indianapolis, IN, USA). All measurements were performed in duplicate, with appropriate quality control samples included to ensure analytical precision and reproducibility.

The HOMA-IR index was calculated according to the following formula:

$$HOMA-IR=\frac{Fasting\;Insulin\left(\frac{U}{mL}\right)\times Fasting\;Glucose(\frac{mg}{dL})}{405}$$

Pancreatic β-cell function was estimated using the HOMA-β index, calculated as:

$$HOMA-\beta =\frac{20\times Fasting\;Insulin(\frac{U}{mL})}{Fasting\;Glucose\left(\frac{mmol}{L}\right)-3.5}$$

These indices were derived from the original homeostasis model proposed by Matthews et al. [27] and are widely used as validated surrogate measures of insulin resistance and β-cell function in clinical and metabolic research [28].

### 2.9. Statistical Analysis

Statistical analyses were conducted in accordance with the CONSORT 2010 guidelines [19] and its 2025 update [29]. The level of statistical significance was set at α = 0.05. Where multiple comparisons were performed, Bonferroni correction was applied as appropriate [30].

Normality of continuous variables was assessed using the Shapiro–Wilk test [31]. As the assumption of normality was not met for most variables (*p* < 0.05), non-parametric statistical methods were used throughout. Continuous data are therefore presented as median and interquartile range (IQR) [32].

Between-group comparisons (EDC-HHA01 vs. placebo) at individual time points (baseline or month 6) were performed using the Mann–Whitney U test. Within-group changes between baseline and month 6 were assessed using the Wilcoxon signed-rank test [32]. Longitudinal analyses across three time points (baseline, month 3, and month 6) were conducted using the Friedman test, followed by Bonferroni-corrected post hoc comparisons.

Comparisons among the four clinical subgroups (T2DM + placebo, T2DM + EDC-HHA01, PD + placebo, PD + EDC-HHA01) were performed using the Kruskal–Wallis test, with Dunn’s post hoc tests applied where appropriate. Stratified analyses by baseline diagnosis (T2DM vs. PD were conducted with adjustment of the significance threshold to α = 0.0083 (0.05/6) to account for multiple subgroup comparisons.

Adverse events and perceived benefits, assessed using a Likert scale (1–5), were analyzed using the Mann–Whitney U test for between-group comparisons and the Wilcoxon signed-rank test for within-group changes. Bonferroni correction was applied for analyses involving 30 questionnaire items (adjusted α = 0.0017) [30,32].

Effect sizes for non-parametric tests were calculated using Pearson’s r (r = 0.10, small; r = 0.30, moderate; r = 0.50, large). For variables meeting assumptions of homogeneity of variance (assessed using Levene’s test), Cohen’s d was calculated; where variance assumptions were violated, Welch’s corrected Cohen’s d was applied [33].

All statistical analyses were performed using SPSS version 27.0 (IBM Corp., Armonk, NY, USA) and Python v3.10.8 [34], with data visualization conducted using Matplotlib [35] and REDC-HHA01y v1.0 packages [36] within the Google Colab environment [37]. Anonymized datasets and analytical code will be made available upon reasonable request to the corresponding author, in accordance with institutional ethics approval and data transparency policies.

#### 2.9.1. Statistical Effect Size Estimation

In addition to hypothesis testing, effect sizes were calculated to quantify the magnitude of observed differences between treatment groups. For non-parametric comparisons, Cliff’s delta (δ) was used as a distribution-free measure of effect size [38].

Cliff’s delta estimates the probability that a randomly selected observation from one group will be greater than a randomly selected observation from another group, minus the reverse probability. Values range from −1 to +1, where 0 indicates complete overlap between distributions. Positive values indicate higher values in the treatment group, while negative values indicate higher values in the comparator group.

Cliff’s delta was calculated for between-group comparisons of change scores (Δ = 6 months − baseline), including fasting glucose and HbA1c where appropriate. Effect size magnitudes were interpreted using established thresholds: |δ| < 0.147 (negligible), 0.147 ≤ |δ| < 0.33 (small), 0.33 ≤ |δ| < 0.474 (moderate), and |δ| ≥ 0.474 (large).

Within-group effect sizes for paired analyses were estimated using Cohen’s dz, calculated as the mean of paired differences divided by the standard deviation of those differences. Effect size estimates were reported alongside corresponding non-parametric test results to facilitate interpretation of clinical relevance independent of statistical significance.

All effect size calculations were performed using Python-based statistical libraries, and values are reported without confidence intervals given the exploratory nature of subgroup analyses [39]. Data visualization was performed using Python (Matplotlib) [35].

#### 2.9.2. Handling of Missing Data

Missing outcome data were minimal. Analyses were conducted according to the intention-to-treat principle using all available data. Participants with missing measurements for a given outcome were excluded from analyses of that specific outcome only (available-case analysis). No imputation methods were applied due to the low proportion of missing data and the absence of evidence suggesting differential missingness between treatment groups.

##### Additional and Exploratory Analyses

Additional analyses were prespecified to explore treatment effects across clinically relevant subgroups, including stratification by baseline diagnostic category (PD vs. T2DM) and background metformin use. These subgroup analyses were conducted using non-parametric methods consistent with the primary analysis plan and were interpreted cautiously given reduced sample sizes.

Exploratory analyses included evaluation of secondary metabolic endpoints, composite markers of metabolic endotoxemia derived from two independent analytical platforms, and patient-reported outcomes related to gastrointestinal tolerability and perceived benefits. Exploratory analyses were intended to provide supportive and hypothesis-generating insights rather than confirmatory evidence. Where applicable, effect sizes were emphasized alongside *p*-values, and no additional adjustments for multiple comparisons were applied beyond those specified for questionnaire-based analyses.

## 3. Results

### 3.1. Analysis Populations

A total of 92 participants were randomized and included in the intention-to-treat analysis. Participants were stratified a priori by metabolic status into PD and T2DM subgroups. Baseline demographic and clinical characteristics did not differ significantly between intervention and placebo groups within each diagnostic stratum (independent *t*-tests or Mann–Whitney U tests, all *p* > 0.05; Table 1). Study adherence, assessed by capsule count, exceeded 94% across groups, and no protocol deviations affecting the primary outcome analyses were identified.

### 3.2. Baseline Demographic and Clinical Characteristics

Baseline metabolic parameters, including HbA1c, fasting glucose, insulin, and indices of insulin resistance, did not differ significantly between intervention and placebo groups within diagnostic strata (Table 1). Baseline symptom and quality-of-life scores were likewise comparable across groups.

### 3.3. Primary Outcome: Glycemic Control (HbA1c)

**Figure 2:**
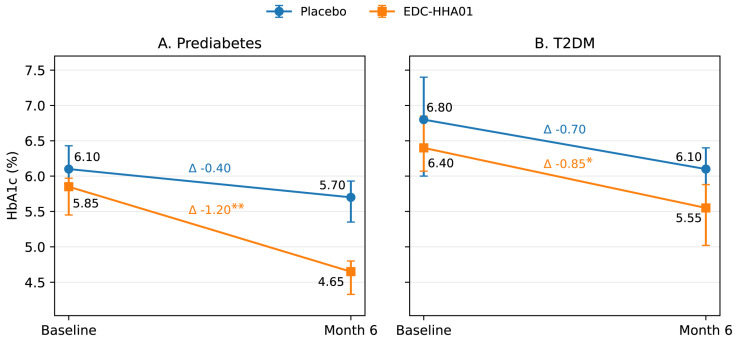
HbA1c trajectories over 6 months by treatment arm and diagnostic category: (**A**) Median HbA1c values with IQR at baseline, month 3, and month 6 for PD subgroups. (**B**) Median HbA1c values for T2DM subgroups. *, ** denote significant within-group change from baseline (Wilcoxon, *p* < 0.05, *p* < 0.01 respectively).

Changes in HbA1c over the intervention period are summarized in Table 2 and Table 3 and illustrated in Figure 2.

In participants with PD the intervention group demonstrated a statistically significant reduction in HbA1c from baseline compared with placebo (Table 2, Figure 2A (median: 5.75%) to month 6 (median: 4.60%), with a median change (Δ) of −1.15% (*p* < 0.001, Wilcoxon signed-rank test). The magnitude of HbA1c reduction was greater in the intervention group, resulting in a clinically meaningful between-group difference at study end (Δ median = −0.55%; *p* = 0.007 for between-group comparison of ΔHbA1c, Mann–Whitney U test), resulting in a clinically meaningful between-group difference at study end (Table 2, Figure 2A). Effect size analysis using Cliff’s delta indicated a large treatment effect favoring EDC-HHA01 in PD (δ = −0.64; indicates a large effect).

In participants with T2DM, HbA1c levels showed a modest reduction in the intervention group (Δ median = −1.00%), but the between-group difference compared to placebo was ((Δ median = −0.70%); Table 2); however, between-group differences did not reach statistical significance (*p* = 0.223, Mann–Whitney U test; Table 2, Figure 2B). Within-group changes were directionally consistent with those observed in the PD subgroup but were attenuated in magnitude.

Placebo groups exhibited small reductions in HbA1c across diagnostic strata (Table 2), consistent with expected trial participation and lifestyle-related effects. The treatment-over-placebo effect was most pronounced in participants with PD (Figure 2B).

Placebo groups exhibited small reductions in HbA1c across diagnostic strata, consistent with expected trial-participation and lifestyle-related effects. The treatment-over-placebo effect was therefore most pronounced in participants with PD.

Stratification by background metformin use showed that the treatment effect on HbA1c was strongest among participants with PD who were not receiving metformin (Table 3).

Figure 2 illustrates median change from baseline to month 6 of HbA1c from baseline to Month 6 in placebo and EDC-HHA01 groups, stratified by diagnostic category (PD and T2DM).

### 3.4. Secondary Glycemic Measurements

#### 3.4.1. Fasting Glucose

No statistically significant between-group differences in fasting glucose were observed in either diagnostic subgroup (Table S2). Variability in fasting glucose measurements was high, and changes were considered supportive rather than primary indicators of intervention efficacy.

#### 3.4.2. Postprandial Glucose (2 h)

Postprandial glucose responses demonstrated directionally favorable trends in the intervention group, particularly among participants with PD; however, these differences did not reach statistical significance (Table S3). Given the exploratory nature of these analyses and the heterogeneity of background glucose-lowering therapies, no additional stratified analyses were performed.

### 3.5. Endotoxemia and Inflammatory Markers

**Figure 3:**
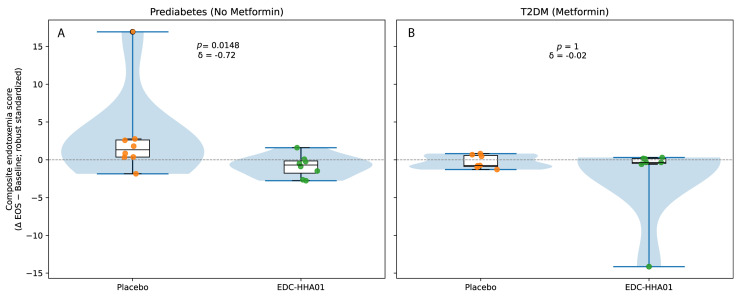
Changes following EDC-HHA01 supplementation stratified by metabolic status and metformin use. Within-subject changes in circulating endotoxemia were calculated as End of Study − Baseline for two independent lipopolysaccharide (LPS) assays (GeneScript/LAL-based assay and ELISA). For each platform, changes were robustly standardized using median and median absolute deviation (MAD), and the resulting standardized values were averaged to generate a composite endotoxemia score for each participant. Lower composite scores indicate greater reductions in endotoxemia. Individual data points are overlaid, with orange dots representing placebo and green dots representing EDC-HHA01. The light blue shaded violin plots depict the kernel density distribution of Δ EOS values within each group. (**A**) Participants with PD not receiving metformin demonstrated a significant treatment-associated reduction in composite endotoxemia scores compared with placebo. (**B**) In participants with established T2DM receiving background metformin therapy, no meaningful separation between treatment and placebo groups was observed. Raincloud plots [40] display kernel density distributions (half violins), boxplots show medians and interquartile ranges, and individual participant datapoints. Horizontal dashed lines denote no change (Δ = 0). Between-group comparisons were performed using the Mann–Whitney U test, with effect sizes quantified by Cliff’s δ and annotated within each panel.

Markers of metabolic endotoxemia are summarized in Table 4 and visualized in Figure 3. Descriptive distributions of the composite endotoxemia change scores indicate opposing median shifts between treatment groups in participants with PD (Figure 3A,B). Specifically, placebo-treated participants with PD exhibited a positive median change consistent with increased endotoxemia over the study period, whereas participants receiving EDC-HHA01 demonstrated a negative median change consistent with reduced endotoxemia (Table 4, Figure 3A).

Consistent with these distributional patterns, stratified inferential analyses demonstrated a statistically significant treatment-associated reduction in circulating lipopolysaccharide (LPS) levels in participants with PD receiving EDC-HHA01 compared with placebo, with effect size estimates in the moderate range (*p* < 0.05; Table 4). In contrast, among participants with established T2DM, composite endotoxemia changes were modest and overlapping between treatment groups, indicating attenuated separation in later-stage disease.

In the T2DM subgroup, reductions in LPS were directionally similar but did not reach statistical significance.

Overall, reductions in metabolic endotoxemia were most evident among participants with earlier-stage metabolic dysregulation.

Visual representations of these changes are shown in Figure 3.

### 3.6. Insulin Resistance and β-Cell Function

Indices of insulin resistance and β-cell function are summarized in Table 4.

In participants with PD, the intervention group (G4) demonstrated statistically significant between-group superiority in HOMA-IR change scores compared with placebo (G3) (Δ between-group *p* = 0.042; Mann–Whitney U test of change scores; Table 5). Reductions in fasting insulin and associated derived indices were directionally consistent with the pattern of glycemic improvement observed in this subgroup (Table 5).

In participants with T2DM, HOMA-IR change scores did not differ significantly between intervention (G2) and placebo (G1) groups (Δ between-group *p* = 0.387; Table 5).

A comprehensive summary of insulin-related and additional metabolic outcomes is provided in Table S2.

### 3.7. Other Secondary Metabolic Outcomes

Changes in renal function and lipid parameters were evaluated as secondary and safety-related outcomes (Table S1). Modest within-group changes were observed in select lipid measures and creatinine; however, these changes were not consistent across diagnostic or treatment strata and did not demonstrate a clear treatment-over-placebo effect. All measured values remained within clinically acceptable reference ranges over the 6-month intervention period.

### 3.8. Safety and Tolerability

Safety and tolerability were evaluated over the six-month intervention period using a structured patient-reported survey comprising 36 predefined symptoms and perceived adverse effects (Table S4). Responses were recorded on a 5-point ordinal scale and analyzed using non-parametric methods due to non-normal distributions observed for several variables.

No serious adverse events occurred. EDC-HHA01 showed excellent tolerability with >94% adherence; modest GI symptom improvements vs. placebo (gas/bloating, diarrhea, constipation; Supplementary Table S3).

Across the full symptom inventory, within-group analyses (Wilcoxon signed-rank test) demonstrated modest improvements over time in several gastrointestinal and general well-being-related symptoms in both groups (Table S4). Between-group comparisons at study end (Mann–Whitney U test) indicated that participants receiving EDC-HHA01 experienced greater reductions in the frequency and severity of selected gastrointestinal complaints, including gas/bloating, diarrhea, and constipation, compared with placebo (*p* < 0.05; Table 6. These findings were associated with small-to-moderate effect sizes (Cliff’s δ = 0.32–0.47; Table 5) and are consistent with a favorable tolerability profile rather than a therapeutic effect.

Additional between-group differences favoring EDC-HHA01 were observed for headache frequency and sleep disturbance. No significant differences between groups were detected for dermatologic, respiratory, or other non-gastrointestinal symptoms. Effect sizes for statistically relevant comparisons were generally small to moderate and consistent with a favorable tolerability profile rather than a therapeutic effect.

Given the exploratory nature of patient-reported outcomes and the number of comparisons performed, these findings should be interpreted as supportive evidence of tolerability and acceptability rather than confirmatory efficacy signals. Detailed results for all monitored symptoms and perceived adverse effects are presented in Table S3.

### 3.9. Patient-Reported Perceived Benefits (Exploratory Analysis)

An exploratory analysis of patient-reported perceived benefits was conducted to characterize subjective changes in gastrointestinal comfort, appetite-related perceptions, psychological well-being, and general vitality over the six-month intervention period (Supplementary Table S4). Responses were collected using a structured ordinal questionnaire and analyzed using non-parametric methods.

Compared with placebo, participants receiving EDC-HHA01 reported more favorable perceived changes across several gastrointestinal domains, including reduced intestinal gas (*p* = 0.03), improved bowel habits (*p* = 0.02), and improved stool consistency (*p* = 0.04; Supplementary Table S4). Differences favoring the intervention group were also observed for perceived hunger regulation, digestion-related comfort, and gastric tolerance (Supplementary Table S4).

Participants receiving EDC-HHA01 additionally reported higher perceived physical energy (*p* = 0.02) and lower levels of anxiety at study end relative to placebo (*p* = 0.03; Supplementary Table S4). Across assessed domains, between-group differences were associated with effect sizes in the moderate-to-large range (Cliff’s δ = 0.45–0.68; Supplementary Table S4). Given the subjective nature of these outcomes, the exploratory intent of the analysis, the absence of adjustment for multiple comparisons, and the potential influence of expectation effects, these findings should be interpreted as descriptive of patient experience and acceptability rather than as confirmatory evidence of clinical efficacy.

## 4. Discussion

### 4.1. Study Design, Conduct, and Internal Validity

The present study was designed and conducted to address several methodological limitations that have historically contributed to inconsistent or inconclusive findings in probiotic and nutraceutical research [41,42,43]. The randomized, double-blind, placebo-controlled design, combined with predefined inclusion criteria and a six-month intervention period, provided a robust framework for evaluating metabolic outcomes while minimizing bias and expectancy effects.

Randomization and blinding were maintained throughout the study, and baseline demographic and metabolic characteristics were comparable between intervention and placebo groups within diagnostic strata. Adherence to the intervention was high (>94%), as assessed by capsule count, indicating good compliance and supporting the reliability of the observed outcomes. Importantly, no protocol deviations affecting primary or secondary analyses were identified.

Following randomization, all participants were included in the intention-to-treat population. Losses to follow-up and exclusions after randomization were minimal and occurred at similar frequencies in the EDC-HHA01 and placebo groups. Reasons for loss included missed follow-up visits, inability to provide biological samples at scheduled time points, or withdrawal of consent for personal reasons. No participants were excluded due to adverse events or protocol-defined safety concerns. The number of participants contributing data to each analysis is reported in the corresponding tables and figures.

Outcome measures were selected a priori and included both objective biomarkers (e.g., HbA1c, insulin resistance indices [44], endotoxemia markers) and patient-reported assessments. Objective metabolic endpoints were analyzed using non-parametric statistical approaches appropriate for the distribution of the data, and effect sizes were reported alongside *p*-values to provide context for the magnitude of observed differences [45]. Exploratory analyses revealed additional within-group improvements across several cardiometabolic parameters (e.g., total cholesterol, LDL, Castelli index; Supplementary Table S1), suggesting potential broader benefits of EDC-HHA01 that warrant investigation in future hypothesis-driven studies. Exploratory analyses were clearly distinguished from primary and secondary endpoints to avoid overinterpretation [46].

Safety and tolerability were evaluated systematically using a structured symptom inventory, and comprehensive reporting of adverse effects was maintained [47]. No serious adverse events were reported, and no participants discontinued the study due to tolerability concerns. The absence of clinically meaningful changes in renal or lipid parameters further supports the internal validity and safety profile of the intervention over the study period [48].

Collectively, these design and conduct features support the internal validity of the trial and provide confidence that the observed metabolic and biological effects are unlikely to be attributable to methodological artifacts or differential compliance.

### 4.2. Glycemic Control and Stage-Dependent Effects on HbA1c

The primary metabolic finding of this study was a statistically and clinically meaningful reduction in HbA1c among participants with PD receiving EDC-HHA01. This effect was consistently observed across analytical approaches, including within-group comparisons and between-group treatment effect analyses, and was supported by moderate-to-large effect sizes in participants not receiving background metformin therapy. In contrast, reductions in HbA1c among participants with established T2DM were directionally similar but attenuated and did not yield statistically significant treatment-over-placebo effects.

The magnitude of HbA1c reduction observed in PD is notable given the non-pharmacologic nature of the intervention and the six-month duration of supplementation. HbA1c reflects integrated glycemic exposure over approximately 8–12 weeks and is less sensitive to short-term behavioral fluctuations than fasting or postprandial glucose measurements. Consequently, changes in HbA1c are generally interpreted as reflecting sustained alterations in glucose handling rather than transient effects related to study participation alone [49,50]. The observation that placebo groups exhibited modest HbA1c reductions is consistent with known trial participation and lifestyle effects [51,52,53]; however, the greater magnitude of change and treatment-over-placebo separation in the PD subgroup receiving EDC-HHA01 suggests a biologically relevant intervention effect.

Stratification by background metformin use provides additional context for these findings. Among participants with PD not receiving metformin, the treatment effect on HbA1c was strongest, whereas participants already receiving metformin exhibited smaller and more variable changes. This pattern is consistent with the established glucose-lowering efficacy of metformin and suggests that the incremental impact of a microbiome-targeted intervention may be more readily detectable in individuals not already receiving pharmacologic therapy [54,55].

Post hoc analyses revealed metformin use significantly attenuated EDC-HHA01 efficacy (HbA1c Δ between-group *p* = 0.023; n = 44 metformin vs. n = 31 non-metformin). This interaction likely reflects metformin’s microbiome-modifying effects, particularly its robust promotion of *Akkermansia muciniphila* [56,57]. While physiological *Akkermansia* levels strengthen the mucin barrier, enhance tight junction integrity, and boost SCFA production via gut–liver crosstalk [56], pharmacological overabundance—as potentially induced by chronic metformin—may deplete the mucus layer, increasing lipopolysaccharide leakage and inflammation [58].

Animal models confirm *Akkermansia* mediation of metformin’s antidiabetic effects [59], suggesting saturation of microbial niches in metformin-treated T2DM patients. Our ongoing microbiome analyses from this cohort will test whether EDC-HHA01’s *Bifidobacterium/Lactobacillus* consortium elicits competitive exclusion of metformin-expanded *Akkermansia*, or alternatively, whether sequential administration (metformin → synbiotic) could optimize therapeutic synergy. These findings highlight disease-stage specificity: prediabetes patients retain microbiome plasticity for synbiotic benefits (HOMA-IR δ = 0.62), while advanced T2DM exhibits microbial rigidity potentially exacerbated by metformin–*Akkermansia* interactions.

Importantly, the study was not designed or powered to evaluate add-on effects to metformin, and conclusions regarding combination effects should therefore be interpreted cautiously.

The attenuated response observed in participants with T2DM likely reflects greater metabolic heterogeneity, longer disease duration, and more advanced impairments in insulin sensitivity and β-cell function. Prior studies have demonstrated that microbiome composition, intestinal barrier integrity, and host–microbe signaling pathways are progressively altered with advancing metabolic disease, potentially limiting the responsiveness of later-stage T2DM to interventions targeting microbial or barrier-related mechanisms [6,8,60]. In this context, the present findings are consistent with the concept that microbiome-targeted strategies may exert greater influence during earlier stages of metabolic dysregulation, when host regulatory pathways remain more plastic [61,62,63].

Collectively, these HbA1c findings support the interpretation that EDC-HHA01 provides metabolic support that is most evident in PD rather than functioning as a glucose-lowering therapy in established T2DM. This stage-dependent pattern aligns with current understanding of metabolic disease progression and underscores the importance of considering disease context when evaluating non-pharmacologic interventions aimed at modulating host–microbe interactions [11,64,65].

### 4.3. Biological Coherence: Endotoxemia and Insulin Resistance

The observed improvements in glycemic control in participants with PD were accompanied by reductions in circulating markers of metabolic endotoxemia and favorable changes in indices of insulin resistance, providing biological context for the primary HbA1c findings. While the present study was not designed to establish mechanistic causality, the convergence of these outcomes supports a coherent interpretation of the metabolic effects observed.

Metabolic endotoxemia, commonly assessed by circulating lipopolysaccharide (LPS) levels, has been implicated in the development of low-grade systemic inflammation and impaired insulin signaling [8,66,67,68]. Experimental and clinical studies have demonstrated that chronic exposure to endotoxin can activate innate immune pathways, disrupt insulin receptor signaling, and contribute to metabolic dysregulation, particularly in early stages of insulin resistance [8,69,70]. In the present study, reductions in LPS-related measures were most evident in participants with PD receiving EDC-HHA01, whereas changes in participants with established T2DM were smaller and did not reach statistical significance.

Improvements in fasting insulin levels and HOMA-derived indices observed in the PD subgroup further align with this pattern. Insulin resistance is a central feature of early metabolic dysfunction and is sensitive to inflammatory and barrier-related perturbations. Prior work has shown that interventions associated with reduced endotoxin exposure or improved intestinal barrier function are frequently accompanied by improvements in insulin sensitivity, even in the absence of marked changes in fasting glucose [6,71,72]. The directional concordance between reduced endotoxemia markers and improved insulin resistance indices in this study is therefore consistent with established biological relationships.

The stage-dependent nature of these findings is notable. In PD host–microbe interactions, intestinal permeability, and inflammatory signaling pathways may retain greater functional plasticity, rendering them more responsive to interventions targeting microbial or barrier-associated processes [73,74,75]. In contrast, advanced T2DM is characterized by greater heterogeneity, longer disease duration, medication effects, and more entrenched impairments in insulin signaling and β-cell function, factors that may limit responsiveness to microbiome-targeted strategies [11,60,76,77]. This framework provides a plausible explanation for the attenuated biological responses observed in participants with T2DM.

Importantly, the present findings should not be interpreted as evidence that reductions in endotoxemia directly caused improvements in glycemic control. Rather, the parallel changes observed across endotoxemia markers, insulin resistance indices, and HbA1c suggest that these outcomes may reflect interrelated aspects of a broader metabolic response. Such convergence strengthens confidence in the biological plausibility of the observed effects without exceeding the evidentiary scope of the study.

Taken together, the alignment of glycemic, insulin-related, and endotoxemia-associated outcomes supports a biologically coherent interpretation of the metabolic effects of EDC-HHA01 in individuals with PD [67,68,74]. These findings are consistent with current models linking host–microbe interactions, inflammatory tone, and insulin sensitivity in early metabolic dysregulation, and they further reinforce the importance of disease stage in evaluating microbiome-targeted interventions [6,8,11,60].

### 4.4. Patient Experience and Tolerability in Context

Patient-reported outcomes assessing perceived gastrointestinal comfort, appetite-related perceptions, psychological well-being, and general vitality were evaluated as exploratory measures to contextualize participant experience during the intervention. These outcomes were not prespecified as efficacy endpoints and were analyzed conservatively, with full results provided in Supplementary Table S4.

Participants receiving EDC-HHA01 reported more favorable perceived changes across several gastrointestinal domains, including reduced intestinal gas, improved bowel habits, and improved stool consistency, relative to placebo. Additional differences favoring the intervention group were observed for perceived digestion-related comfort, hunger regulation, physical energy, and anxiety levels at study end. These findings were associated with moderate-to-large effect sizes but were not adjusted for expectation effects or multiple comparisons.

Given the subjective nature of these assessments, these outcomes should not be interpreted as confirmatory evidence of metabolic efficacy. Patient-reported measures are inherently sensitive to placebo responses, expectancy bias, and contextual factors, particularly in nutrition and microbiome studies [78,79,80,81]. Accordingly, these findings are best viewed as descriptive indicators of participant experience rather than as primary evidence of biological effect.

Importantly, the favorable patient-reported profile observed in the intervention group aligns with the high adherence and favorable tolerability documented over the six-month study period. In this context, perceived improvements in gastrointestinal comfort and general well-being may contribute to sustained engagement with the intervention, a factor of practical relevance in long-term metabolic health strategies [82,83,84]. Prior studies have noted that tolerability and subjective comfort can meaningfully influence adherence [85] to non-pharmacologic interventions, even when objective metabolic endpoints are modest [80,86].

When considered alongside objective metabolic and biological outcomes, patient-reported findings provide contextual support rather than independent validation. Improvements in HbA1c, insulin resistance indices, and endotoxemia markers offer biological grounding for the intervention, while patient-reported outcomes help characterize acceptability and real-world feasibility [85,87]. This triangulated interpretation is consistent with best practices in clinical nutrition and microbiome research, where subjective experience and objective biomarkers are complementary but not interchangeable sources of evidence [88].

### 4.5. Safety and Metabolic Neutrality

Safety and metabolic neutrality are critical considerations in the evaluation of non-pharmacologic interventions intended for long-term use [89]. In the present study, EDC-HHA01 was well tolerated over the six-month intervention period, with no serious adverse events reported and no discontinuations attributable to safety concerns. Overall adherence exceeded 94%, further supporting the acceptability of the intervention.

Systematic assessment of adverse effects using a structured symptom inventory did not reveal any clinically concerning safety signals. Between-group comparisons indicated either no differences or modest improvements in selected gastrointestinal tolerability measures favoring the intervention group. In addition, exploratory patient-reported outcomes demonstrated greater perceived improvements in multiple gastrointestinal domains—including bowel habits, stool consistency, intestinal gas, and gastric comfort—among participants receiving EDC-HHA01 compared with placebo. Although these outcomes were subjective and not adjusted for multiple comparisons, the consistency and magnitude of the observed differences support a favorable tolerability and acceptability profile rather than a therapeutic effect.

Notably, several of the gastrointestinal domains showing improvement overlap with symptoms commonly reported in association with metformin use, including diarrhea and altered bowel habits [90,91]. While the present study was not designed or powered to evaluate metformin-associated gastrointestinal effects and outcomes were not formally stratified by background metformin therapy, these exploratory observations are biologically plausible given known interactions between metformin, the gut microbiome, bile acid metabolism, and intestinal barrier function [92,93]. Accordingly, these findings should be interpreted cautiously but suggest that microbiome-targeted interventions may merit further investigation for their potential to improve gastrointestinal tolerability in individuals receiving glucose-lowering medications.

Evaluation of secondary metabolic and safety-related laboratory parameters further demonstrated metabolic neutrality across key domains. Renal function markers and lipid parameters remained within clinically acceptable reference ranges throughout the study, and changes observed over time were modest, inconsistent across strata, and did not demonstrate a clear treatment-over-placebo effect. These findings indicate that the observed improvements in glycemic and insulin-related outcomes were not accompanied by off-target metabolic perturbations.

Taken together, the safety and laboratory findings support the conclusion that EDC-HHA01 can be administered over a six-month period without evidence of adverse metabolic effects. This favorable safety and tolerability profile provides an important foundation for the interpretation of the metabolic findings and supports further investigation of microbiome-targeted strategies in early metabolic dysregulation [76,94].

### 4.6. Study Limitations

Several limitations of the present study should be acknowledged when interpreting the findings. First, although the trial was designed with sufficient statistical power (≥80%) to detect clinically meaningful changes in the primary outcome (HbA1c), the overall sample size limits the precision of subgroup analyses, particularly when stratifying by diagnostic category and background metformin use. Accordingly, findings within smaller strata should be interpreted with appropriate caution.

Importantly, the adequacy of statistical power for the primary endpoint supports the internal validity of the observed HbA1c effects in participants with PD. The consistency of these findings across analytical approaches, including within-group comparisons, between-group treatment effect analyses, and effect size estimates, further reinforces confidence that the primary metabolic signal is unlikely to be attributable to random variation. Larger studies will nevertheless be required to refine effect estimates, assess heterogeneity of response, and evaluate additional modifiers of treatment effect.

Second, although the study incorporated multiple objective metabolic and biological endpoints, direct characterization of gut microbiome composition and functional capacity was not included in the present analysis. Microbiome sequencing and associated functional data have been generated and are currently undergoing independent analysis. Given the complexity of the clinical dataset and the importance of maintaining analytical clarity, the present manuscript was intentionally focused on clinical outcomes, metabolic biomarkers, and patient-reported measures. Detailed microbiome and mechanistic analyses will be reported separately to allow appropriate depth of interpretation.

Third, participants with established T2DM exhibited greater heterogeneity in disease duration, background therapy, and metabolic status, which may have reduced sensitivity to detect treatment-over-placebo effects in this subgroup. The study was not designed to evaluate add-on efficacy in combination with glucose-lowering medications, and conclusions regarding such use should therefore be avoided.

Fourth, patient-reported outcomes were exploratory in nature and inherently subject to expectation effects and placebo responses. These measures were included to contextualize participant experience and tolerability rather than to establish efficacy and should be interpreted accordingly.

Finally, the current study lacks detailed data on lifestyle factors, such as diet and physical activity, which could influence glycemic parameters and explain the improvements observed in the placebo group. In future research, it is essential to account for these covariates.

Taken together, these limitations reflect constraints of study scope rather than deficiencies in study conduct. The findings provide internally valid evidence supporting a stage-dependent metabolic effect of EDC-HHA01 in PD while underscoring the need for larger, mechanistically informed trials to confirm generalizability, explore longer-term outcomes, and integrate microbiome-derived insights.

### 4.7. Implications and Future Directions

The findings of the present study have several implications for the evaluation and development of microbiome-targeted strategies in metabolic health. The demonstration of a statistically and clinically meaningful improvement in HbA1c among individuals with prediabetes (PD), supported by concordant changes in insulin resistance indices and markers of metabolic endotoxemia, suggests that non-pharmacologic interventions may exert their greatest impact during earlier stages of metabolic dysregulation. This stage-dependent pattern reinforces the importance of aligning intervention strategies with disease context rather than extrapolating effects across heterogeneous metabolic states.

From a clinical perspective, these results support further investigation of microbiome-informed approaches as adjunctive metabolic support strategies, rather than as substitutes for established pharmacologic therapies in overt disease. The favorable safety and tolerability profile observed over six months, together with high adherence, underscores the feasibility of longer-term use in populations at risk for progression to type 2 diabetes mellitus (T2DM). Such characteristics are particularly relevant in early intervention paradigms, where acceptability and sustained engagement are critical determinants of real-world effectiveness.

An important next step is the evaluation of EDC-HHA01 in drug-naïve individuals with prediabetes, in whom glycemic trajectories—particularly changes in HbA1c, fasting glucose, and insulin resistance—can be assessed without the confounding influence of background glucose-lowering medications. Studies in this population would allow clearer attribution of metabolic effects, refine estimates of treatment magnitude, and help determine whether early microbiome-targeted intervention can meaningfully alter progression toward pharmacologic dependence.

Future studies should therefore prioritize larger, prospectively powered trials designed to refine effect estimates, evaluate durability of response, and assess heterogeneity across demographic and metabolic subgroups. Integration of comprehensive microbiome profiling, functional metagenomics, and metabolomic analyses will be essential to elucidate the biological mechanisms underlying the observed clinical effects and to identify predictors of response. In addition, studies explicitly designed to evaluate interactions with background therapies, including glucose-lowering medications, may further clarify appropriate clinical use contexts.

In summary, the present findings provide internally valid clinical evidence supporting a biologically coherent, stage-dependent metabolic effect of EDC-HHA01 in individuals with prediabetes. By combining objective metabolic biomarkers with careful assessment of tolerability and patient experience, this work establishes a foundation for subsequent mechanistic investigations and larger-scale clinical studies aimed at advancing microbiome-informed strategies for metabolic health.

## 5. Conclusions

In this randomized, double-blind, placebo-controlled clinical trial, six months of supplementation with EDC-HHA01 was associated with a statistically and clinically meaningful improvement in glycemic control among individuals with PD. These effects were supported by concordant changes in insulin resistance indices and markers of metabolic endotoxemia, providing biological coherence to the observed HbA1c reductions. In contrast, metabolic responses in participants with established T2DM were attenuated, underscoring the importance of disease stage in evaluating microbiome-targeted interventions.

The study was adequately powered for its primary endpoint and demonstrated high adherence and a favorable safety and tolerability profile, supporting the internal validity of the findings. Secondary and exploratory outcomes, including patient-reported measures, contributed contextual insight into acceptability and feasibility but were not interpreted as confirmatory efficacy signals.

Collectively, these findings support the potential relevance of microbiome-informed, non-pharmacologic strategies as metabolic support in early-stage dysregulation rather than as glucose-lowering therapies in advanced disease. Larger, mechanistically informed trials incorporating comprehensive microbiome analyses will be required to confirm generalizability, refine responder profiles, and further elucidate underlying biological pathways.

## 6. Patents

A provisional patent application related to the compositions and methods described in this study has been filed.

## Figures and Tables

**Table 1:** Baseline distribution of epidemiological variables by treatment group.

Variables		Group No (%)		Total	*p*
		Placebo	EDC-HHA01		
Sex	Female	28 (75.7)	21 (55.3)	49 (65.3)	0.106 ^a^
	Male	9 (24.3)	17 (44.7)	26 (34.6)	
Diagnosis	T2DM	21 (56.8)	20 (52.6)	41 (54.7)	0.899 ^a^
	PD	16 (43.2)	18 (47.4)	34 (45.3)	
Treatment *	No	15 (40.6)	16 (42.1)	31 (41.3)	1.000 ^a^
	Yes	22 59.5)	22 (57.9)	44 (58.7)	
Physical Activity	No	17 (45.9)	17 (44.7)	34 (45.3)	1.000 ^a^
	Yes	20 (54.1)	21 (55.3)	41 (54.7)	
Age (mean ± SD)		59.9 ± 12.3	55.6 ± 10.8	57.7 ± 11.7	0.114 ^b^
BMI (mean ± SD)		28.7 ± 6.2	29.5 ± 5.9	29.1 ± 6.0	0.538 ^b^

^a^ Refers to the Chi-square test; ^b^ Refers to Student’s *t*-test; * Refers to whether the patient is undergoing treatment with oral hypoglycemic agents for the metabolic condition (Diabetes or PD).

**Table 2:** Changes in HbA1c from baseline to 6 months stratified by diagnosis and metformin use.

Cohort	Diagnosis	Metformin	n	Baseline HbA1c (%) Median (IQR)	6-Month HbA1c (%) Median (IQR)	Δ Median	*p* (Within; Wilcoxon)
Placebo	PD	No	12	6.10 (5.85–6.43)	5.80 (5.57–6.00)	−0.4	0.011
	PD	Yes	4	6.10 (5.78–6.50)	5.05 (4.88–5.33)	−0.8	0.125
	T2DM	No	3	6.80 (6.70–7.30)	6.40 (6.30–6.40)	−0.4	0.25
	T2DM	Yes	18	6.80 (5.85–7.33)	5.95 (5.30–6.70)	−0.7	0.0003
EDC-HHA01	PD	No	13	5.90 (5.70–5.90)	4.60 (4.40–4.70)	−1.3	0.0005
	PD	Yes	5	5.60 (5.30–6.00)	4.80 (4.30–4.80)	−0.5	0.062
	T2DM	No	1	7.00 (7.00–7.00)	5.60 (5.60–5.60)	−1.4	-
	T2DM	Yes	19	6.40 (6.05–6.65)	5.50 (4.95–5.95)	−1	<0.0001

Values are median (IQR). Δ Median reflects within-group change from baseline to 6 months. *p* (within) from Wilcoxon signed-rank test. For n < 2, Wilcoxon is not applicable (-).

**Table 3:** Between-group treatment effect on ΔHbA1c (EDC-HHA01 vs. Placebo) within strata.

Diagnosis	Metformin n	Placebo n	EDC-HHA01 n	Cliff’s δ on Δ (Treated vs. Placebo)	*p* (Between; Mann–Whitney on Δ)
PD	No	12	13	−0.64	0.007
PD	Yes	4	5	0.10	0.901
T2D	No	3	1	−1.00	0.346
T2D	Yes	18	19	−0.24	0.223

**Table 4:** Distribution of composite endotoxemia change by diagnosis and treatment group.

Diagnosis	Cohort	Median	Q1	Q3	Δ Median	Value *p* (Mann–Whitney U)
T2DM	Placebo	−0.75	−0.87	0.57	0.41	0.265
T2DM	EDC-HHA01	−0.34	−0.46	0.16		
PD	Placebo	1.33	0.36	2.63	−2.02	< 0.001
PD	EDC-HHA01	−0.69	−1.77	−0.15		

Values represent the distribution of a robust composite endotoxemia change score calculated as the within-subject difference (End of Study − Baseline) in circulating lipopolysaccharide (LPS) measured by two independent platforms (GeneScript/LAL-based assay and ELISA), standardized using median and median absolute deviation (MAD) and averaged per participant. Negative values indicate greater reductions in endotoxemia. IQRs already present (Q1–Q3 columns). This table is intended to provide descriptive distributional context; stratified inferential analyses accounting for metformin use are presented in Figure 3.

**Table 5:** Glycemic and insulin-related outcomes over 6 months.

Parameter	Group	Baseline Median (IQR)	6-Month Median (IQR)	Δ Median	*p* (Wilcoxon)
Insulin (µIU/mL)	G1	15.0 (10.5–21.5)	9.0 (5.0–17.5)	−6.0	0.001
	G2	19.5 (11.8–27.5)	13.5 (12.1–19.0)	−6.0	0.003
	G3	19.0 (11.2–24.7)	11.0 (5.2–14.5)	−8.0	0.007
	G4	21.0 (16.5–33.5)	11.5 (6.8–20.2)	−9.5	0.001
HOMA-IR	G1	4.7 (2.8–6.7)	2.9 (1.5–5.1)	−1.8	0.002
	G2	4.6 (2.3–8.4)	4.3 (2.4–6.3)	−0.3	0.059
	G3	3.9 (2.4–6.0)	2.4 (1.3–3.4)	−1.5	0.031
	G4	4.9 (3.5–7.8)	3.2 (1.8–5.2)	−1.7	0.020
HOMA-β	G1	13.5 (6.9–21.6)	8.3 (3.4–16.1)	−5.2	0.002
	G2	21.8 (12.8–36.7)	9.5 (7.2–15.4)	−12.0	0.001
	G3	28.2 (17.9–52.6)	11.3 (6.6–27.7)	−16.9	<0.001
	G4	46.9 (20.8–100.2)	15.1 (8.3–22.1)	−31.8	<0.001

G1 (T2DM + Placebo); G2 (T2DM + EDC-HHA01); G3 (PD + Placebo); G4 (PD + EDC-HHA01) Median (IQR). *p* within: Wilcoxon signed-rank (within-group). *p* between: Mann–Whitney U (change scores, intervention vs. placebo within diagnosis).

**Table 6:** Selected adverse events and tolerability outcomes.

Symptom	Direction of Change (EDC-HHA01 vs. Placebo)	Effect Size † (r)	*p*-Value (Mann–Whitney U)	Tolerability Interpretation
Gas/bloating	↓ EDC-HHA01 > placebo	0.32	<0.001	Improved GI tolerability
Diarrhea	↓ EDC-HHA01 > placebo	0.39	<0.001	Improved GI tolerability
Constipation	↓ EDC-HHA01 > placebo	0.51	<0.001	Improved GI tolerability
Headache	↓ EDC-HHA01 > placebo	0.20	0.011	Favorable tolerability
Sleep disturbance	↓ EDC-HHA01 > placebo	0.27	<0.001	Favorable tolerability

† Effect sizes are reported as Pearson’s r derived from Mann–Whitney U statistics. Direction of change reflects relative differences between intervention and placebo groups at study end. This table presents selected outcomes for descriptive purposes. Given the exploratory nature of patient-reported measures and the number of comparisons performed, these findings should be interpreted as indicators of tolerability and acceptability rather than confirmatory efficacy outcomes. Downward arrows (↓) indicate a reduction in symptom frequency or severity in the EDC-HHA01 group relative to placebo.

## Data Availability

The original contributions presented in this study are included in the article/Supplementary Materials. Further inquiries can be directed to the corresponding author.

## Supplementary Materials

The following supporting information can be downloaded at: https://www.mdpi.com/article/10.3390/microorganisms14040749/s1, Table S1: Baseline Metabolic Parameters by Study Group and Diagnostic Stratum; Table S2: Comprehensive summary of metabolic, Biochemical and Glycemic parameters Across study groups; Table S3: Exploratory postprandial Glucose responses by intervention group; Table S4: Participant-Reported Adverse Effects and Benefits: Comparison Between Intervention and Placebo Groups.
