# Supplementary material for: Effects of the Synbiotic Formulation EDC-HHA01 on Glucose Regulation in Adults with Type 2 Diabetes and Prediabetes: A Randomized, Placebo-Controlled Study
Source: Microorganisms. 2026 Mar 26;14(4):749. doi: 10.3390/microorganisms14040749 (PMC13118368; doi:10.3390/microorganisms14040749)
Supplement: Supplementary file 1 [file microorganisms-14-00749-s001.zip › microorganisms-4106936-supplementary.pdf]

Table S1. Baseline Metabolic Parameters by Study Group and Diagnostic Stratum.

Baseline	Placebo DM2 (n=22) Median (IQR)	EDC-HHA01 DM2 (n=18) Median (IQR)	Valor p (DM2)	Placebo PD (n=16) Median (IQR)	EDC-HHA01 PD (n=17) Median (IQR)	Valor p (PD)
Fasting Glucose (mmol/L)	5.76 (4.90 - 7.38)	5.62 (5.00 - 6.12)	0.937	4.77 (4.42 - 5.36)	4.71 (4.38 - 5.12)	0.838
HBA1C1 (%)	6.80 (6.10 - 7.80)	6.40 (6.20 - 7.00)	0.451	6.00 (5.70 - 6.35)	5.80 (5.55 - 6.00)	0.363
Postprandial Glucose (mmol/L)	7.13 (5.69 - 9.85)	6.29 (5.43 - 6.86)	0.271	5.33 (4.68 - 5.69)	4.66 (4.32 - 5.02)	0.246
Insuline (pmol/L)	16.18 (13.70 - 24.97)	19.62 (13.74 - 30.37)	0.638	20.41 (11.52 - 25.46)	24.18 (17.09 - 32.62)	0.266
HOMA	4.8 (3.5 - 7.3)	5.7 (3.2 - 10.6)	0.7	4.3 (2.3 - 6.0)	5.7 (3.5 - 8.8)	0.183
Creatinine (µmol/L)	70.0 (66.2 - 77.0)	76.0 (67.0 - 80.2)	0.484	69.5 (61.0 - 73.5)	81.0 (67.0 - 94.0)	0.043*
Total Cholesterol (mmol/L)	5.03 (4.41 - 5.66)	4.97 (4.04 - 5.90)	0.753	5.33 (4.53 - 6.29)	5.54 (4.79 - 5.85)	0.909
HDL (mmol/L)	1.12 (0.96 - 1.42)	1.00 (0.87 - 1.23)	0.28	1.29 (1.10 - 1.44)	1.21 (0.99 - 1.36)	0.368
LDL (mmol/L)	2.97 (2.52 - 3.69)	2.87 (2.28 - 3.34)	0.877	3.43 (2.56 - 4.17)	3.30 (2.53 - 3.51)	0.729
Triglycerid (mmol/L)	1.28 (0.82 - 1.64)	1.26 (0.84 - 1.83)	0.876	1.14 (0.81 - 1.32)	1.14 (0.83 - 1.41)	0.854

Baseline metabolic parameters showed balanced distribution across intervention and placebo groups within diagnostic strata.

Table S2. Comprehensive summary of metabolic, Biochemical and Glycemic parameters Across study groups.

Parameter (Reference Range)	Group	Baseline Median (IQR)	End-of-Study (6 Month) Median (IQR)	Δ Median	p-Value (Wilcoxon Test)
**A. Renal & General Safety Markers**					
**Creatinine (47.6-113.4 µmol/L)**	G1	70.0 (65.5–79.5)	68.0 (61.7–75.0)	-2	0.107
	G2	76.0 (67.0–81.0)	82.0 (72.9–88.0)	6	**0.002**
	G3	67.0 (60.2–82.2)	72.0 (66.0–82.2)	**5**	**0.002**
	G4	73.7 (67.5–94.2)	76.5 (66.5–91.8)	-2.8	0.172
**B. Lipid Profile**					
**Total Cholesterol (2.81-5.2 mmol/L)**	G1	5.0 (4.4–5.7)	5.1 (4.5–5.3)	0.1	0.796

	G2	5.0 (4.1–6.0)	5.1 (4.4–5.8)	0.1	1.000
	G3	5.2 (5.0–7.0)	5.0 (4.2–6.0)	-0.2	**0.021**
	G4	6.0 (5.0–6.0)	5.5 (4.0–6.0)	-0.5	0.206
**HDL-choresterol (0.9-3.0 mmol/L)**	G1	1.0 (0.8–1.4)	1.0 (0.9–2.0)	0	0.157
	G2	1.0 (0.9–1.23)	1.0 (0.8–1.3)	0	1.000
	G3	1.2 (0.9–1.4)	1.2 (0.9–1.4)	0	0.157
	G4	1.1 (0.94–1.35)	1.0 (0.88–1.19)	-0.1	0.317
**LDL (0.3-4.0 mmol/L)**	G1	2.9 (2.2–3.5)	2.6 (2.2–3.1)	-0.35	**0.001**
	G2	2.7 (2.2–4.0)	2.8 (2.5–3.3)	0.1	0.206
	G3	3.0 (2.5–4.2)	3.0 (2.1–3.6)	0	0.214
	G4	3.0 (2.0–3.3)	3.0 (2.0–3.0)	0	0.206
**Triglycerides (0.46-1.8 mmol/L)**	G1	1.28 (0.80–1.64)	1.25 (0.89–1.44)	-0.03	0.285
	G2	1.28 (0.91–1.68)	1.54 (1.16–1.98)	0.26	0.527
	G3	1.0 (0.7–1.5)	1.0 (0.7–1.8)	0	0.655
	G4	1.1 (0.7–2.0)	1.3 (0.8–1.8)	0.2	0.257
**Castelli**	G1	4.70 (3.3–5.3)	4.30 (3.10–4.7)	-0.4	**0.029**
	G2	5.1 (3.7–5.6)	4.9 (4.0–6.1)	-0.2	0.559

	G3	4.7 (3.5–5.8)	4.2 (3.3–5.3)	-0.5	**0.001**
	G4	4.6 (3.9–5.6)	4.8 (3.8–6.0)	0.2	0.351
**C. Glycemic Control (Primary & Secondary)**					
**HbA1c (2.9-6%)**	G1	6.7 (5.8–7.4)	5.8 (5.2–6.4)	-0.9	**<0.001**
	G2	6.4 (6.0–7.7)	5.5 (5.1–6.1)	-0.9	**<0.001**
	G3	6.0 (5.7–6.4)	5.6 (5.2–5.9)	-0.4	**0.002**
	G4	5.9 (5.6–6.0)	4.7 (4.3–5.2)	-1.2	**<0.001**
**Fasting Insulin (2.6-24.9 UI/ml)**	G1	15.0 (10.5–21.5)	9.0 (5.0–17.5)	-6	**0.001**
	G2	19.5 (11.8–27.5)	13.5 (12.10–19.0)	-6	**0.003**
	G3	19.0 (11.2–24.7)	11.0 (5.2–14.5)	-8	**0.007**
	G4	21.0 (16.5–33.5)	11.5 (6.8–20.2)	-9.5	**0.001**
**HOMA-IR**	G1	4.7 (2.8–6.7)	2.9 (1.5–5.1)	-1.8	**0.002**
	G2	4.6 (2.3–8.4)	4.3 (2.4–6.3)	-0.3	0.059
	G3	3.9 (2.4–6.0)	2.4 (1.3–3.4)	-1.5	**0.031**
	G4	4.9 (3.5–7.8)	3.2 (1.8–5.2)	-1.7	**0.020**
**HOMA-β**	G1	13.5 (6.9–21.6)	8.3 (3.4–16.1)	-5.2	**0.002**
	G2	21.8 (12.8–36.7)	9.5 (7.2–15.4)	-12	**0.001**
	G3	28.2	11.3	-16.9	**<0.001**

		(17.9–52.6)	(6.6–27.7)		
	G4	46.9<br>(20.8–100.2)	15.1<br>(8.3–22.1)	-31.8	**<0.001**
**Fasting Glucose (4.4-7 mmol/L)**	G1	5.76<br>(4.90 - 6.86)	5.68<br>(4.85 - 7.40)	0.08	**0.699**
	G2	5.42<br>(5.00 - 6.29)	5.57<br>(4.93 - 7.11)	-0.15	**0.577**
	G3	4.70<br>(4.55 - 5.00)	4.79<br>(4.39 - 5.37)	-0.09	**0.437**
	G4	4.51<br>(4.31 - 5.03)	4.43<br>(3.91 - 4.83)	0.08	**0.121**

Table S3. Exploratory postprandial Glucose responses by intervention group.

Group	Baseline<br>Median (IQR)	End-of-Study (6 Month)<br>Median (IQR)	Δ Median	p-Valor (Wilcoxon Test)
**G1**	6.27<br>(4.77 - 8.14)	6.48<br>(5.56 - 7.87)	-0.21	0.734
**G2**	5.90<br>(5.07 - 8.06)	6.47<br>(5.73 - 9.17)	-0.57	0.137
**G3**	4.97<br>(4.32 - 5.64)	4.80<br>(4.17 - 5.40)	+0.17	0.297
**G4**	4.49<br>(4.01 - 5.02)	4.87<br>(4.39 - 5.42)	-0.38	0.082

G1: T2DM+placebo; G2: T2DM+ intervention; G3: PD + placebo and G4: PD + intervention.

Table S4. Participant-Reported Adverse Effects and Benefits: Comparison Between Intervention and Placebo Groups.

Variable	EDC-HHA01 Group				Placebo Group				p	Effect Size (Co-
	Mean	SD	Min	Max	Mean	SD	Min	Max	(t-Student)	hen's *d*)
**Q1 general health**	4.18	0.80	2	5	4.06	0.91	2	5	0.579	0.140
**Q2 energy level**	4.18	0.80	2	5	4.10	0.92	2	5	0.707	0.090
**Q3 sleep quality**	3.58	1.06	2	5	3.42	1.26	1	5	0.563	0.140
**Q4 concentration**	4.45	0.80	3	5	4.35	0.85	1	5	0.603	0.130
**Q5 digestion**	4.30	0.79	3	5	4.23	0.81	2	5	0.722	0.090

**Q6 meal regularity**	4.42	0.75	3	5	4.42	0.75	2	5	1.000	0.000
**Q7 reduced hunger**	3.91	1.16	1	5	3.84	1.26	1	5	0.810	0.060
**Q8 craving to snack**	3.70	1.20	1	5	3.77	1.08	2	5	0.794	0.060
**Q9 food cravings**	3.70	1.35	1	5	3.48	1.32	1	5	0.488	0.170
**Q10 appetite easy to control**	3.85	1.32	1	5	3.94	1.24	1	5	0.761	0.080
**Q11 bloating after meals**	4.27	1.04	1	5	3.71	1.6	1	5	0.105	0.410
**Q12 abdominal discomfort or pain**	4.30	1.00	2	5	3.48	1.6	1	5	0.018	0.610
**Q13 bowel movements**	4.42	0.99	2	5	4.19	1.08	1	5	0.352	0.230
**Q14 diarrhea**	4.48	0.80	1	5	3.94	1.31	1	5	0.054	0.490
**Q15 constipation**	4.21	1.22	1	5	4.26	1.17	1	5	0.861	0.040
**Q16 feeling calm**	4.61	0.65	3	5	4.42	0.75	3	5	0.266	0.280
**Q17 stress level**	4.36	0.80	1	5	4.26	0.73	3	5	0.578	0.140
**Q18 positive mood**	4.67	0.60	3	5	4.48	0.63	3	5	0.222	0.310
**Q19 gas**	4.09	0.99	1	5	4.32	0.79	3	5	0.292	0.260
**Q20 general bloating**	4.76	0.50	3	5	4.81	0.4	4	5	0.642	0.120
**Q21 joint pain**	4.94	0.24	4	5	4.84	0.44	3	5	0.249	0.290
**Q22 allergic reaction**	4.97	0.17	4	5	4.87	0.43	3	5	0.238	0.300
**Q23 ease of routine**	4.97	0.17	4	5	4.90	0.30	4	5	0.274	0.280
**Q24 headache**	4.88	0.33	4	5	4.58	0.75	3	5	0.047	0.510
**Q25 infection**	5.00	0.00	5	5	4.94	0.35	2	5	0.347	0.270
**Q26 bronchitis**	5.00	0.00	5	5	4.97	0.18	4	5	0.392	0.240
**Q27 cystitis**	5.00	0.00	5	5	4.97	0.18	4	5	0.392	0.240
**Q28 toothache**	5.00	0.00	5	5	5.00	0.00	5	5	1.000	0.000
**Q29 dizziness**	5.00	0.00	5	5	4.97	0.18	4	5	0.392	0.240
**Q30 muscle discomfort**	5.00	0.00	5	5	5.00	0.00	5	5	1.000	0.000
**Q31 post-capsule pain**	5.00	0.00	5	5	5.00	0.00	5	5	1.000	0.000
**Q32 rhinitis**	5.00	0.00	5	5	4.97	0.18	4	5	0.392	0.240
**Q33 sore throat**	5.00	0.00	5	5	5.00	0.00	5	5	1.000	0.000
**Q34 improved digestion**	4.97	0.17	4	5	4.90	0.40	3	5	0.363	0.230
**Q35 reduced acidity**	4.97	0.17	4	5	4.87	0.43	3	5	0.257	0.290
**Q36 weight reduction**	2.15	1.46	1	5	2.13	1.26	1	5	0.953	0.010
